# Editorial: Food-derived bioactive peptides: preparation, identification, and structure-activity relationship

**DOI:** 10.3389/fnut.2024.1412875

**Published:** 2024-05-02

**Authors:** Shaobo Zhou, Xiao Hu, Lianzhu Lin

**Affiliations:** ^1^School of Science, Faculty of Engineering and Science, University of Greenwich, Chatham, United Kingdom; ^2^Key Laboratory of Aquatic Product Processing, Ministry of Agriculture and Rural Affairs, South China Sea Fisheries Research Institute, Chinese Academy of Fishery Sciences, Guangzhou, China; ^3^School of Food Science and Engineering, South China University of Technology, Guangzhou, China

**Keywords:** isolation, functional applications, identification, biopeptides, food sources

Bioactive peptides form a significant group of low molecular weight protein fragments derived from a wide range of foods, including beans, vegetables, meats, dairy, eggs, seafood, and algae. These peptides exist inactive within the structure of parent proteins until cleaved or are actively produced by microorganisms (1, 2). They offer potential health benefits through antioxidants, cholesterol reduction, thrombosis mitigation, immune response enhancement, antimicrobial resistance, and metal chelation. These attributes have garnered interest in the food, pharmaceutical, and cosmetic industries due to the multifunctionality and excellent biocompatibility.

In this Research Topic, five studies were presented, including analyses on soy peptides (Zhu Y. et al.), calcium chelation (Gu et al.), and antihypertensive peptides (Goyal et al.; Zhu W.-Y. et al.; Li et al.). Soybean products are increasingly recognized for their health benefits and sustainability. They offer protein-rich alternatives for cardiovascular health, obesity management, diabetes control, and lipid metabolism, appealing to diverse dietary preferences, including vegetarian and vegan diets. The sustainability of soybeans enhances their appeal to environmentally conscious consumers. Bioactive peptides derived from soy proteins like glycinin and beta-conglycinin, post-hydrolysis, possess cardiovascular, anti-obesity, diabetes management, and lipid metabolism benefits. Notable among these peptides is lunasin, known for its anti-inflammatory, immunomodulatory effects and potential in cancer prevention (Zhu Y. et al.). Soy peptides, such as lactostatin, play a crucial role in cholesterol and lipid management by inhibiting enzymes like pancreatic lipase and cholesterol esterase, suggesting their role in developing anti-lipidemic products. Their antioxidant properties are vital in reducing oxidative stress and metabolic disorders. The ongoing research into soybean-derived peptides aims at isolating specific bioactive components for targeted health benefits, integrating these peptides into therapeutic strategies and functional foods. This emphasizes their significant role in managing chronic conditions and highlights soybean's potential as a health-promoting agent in future dietary applications.

Three research papers explored peptides derived from moth bean (Goyal et al.), skipjack tuna roes (Zhu W.-Y. et al.), and casein (Li et al.) for their angiotensin-converting enzyme (ACE) inhibitory activity, a crucial factor in regulating blood pressure. The researchers identified peptides with potent ACE inhibitory effects by hydrolyzing these protein concentrates with proteases such as alcalase, papain, and trypsin. The study by Goyal et al. found that alcalase hydrolysis was the most effective, revealing a peptide fraction with significant ACE inhibition. Based on bioinformatic analysis, 15 peptides smaller than 1 kDa were synthesized for their ACE inhibitory potential. Among these, the octapeptide FPPPKVIQ exhibited remarkable inhibitory activity as an uncompetitive inhibitor, maintaining significant effectiveness and stability of the peptide-ACE complex even after simulation of gastrointestinal digestion. This study underscores moth bean as a promising source of natural ACE inhibitors.

Utilizing byproducts from skipjack tuna, Zhu W.-Y. et al.'s study focused on identifying ACE inhibitory peptides and assessing their protective effects against oxidative stress-induced damage in human umbilical vein endothelial cells (HUVECs). Employing flavourzyme for protein hydrolysis, followed by ultrafiltration and chromatographic methods, four ACE-inhibitory peptides were isolated. Molecular docking experiments revealed these peptides' strong binding affinity to ACE, attributable to hydrophobic interactions, electrostatic forces, and hydrogen bonds, highlighting their considerable inhibitory capability. These peptides enhanced nitric oxide production and reduced endothelin-1 secretion in HUVECs, counteracting the adverse effects of norepinephrine and mitigating oxidative damage and apoptosis rates in H_2_O_2_-induced HUVECs by increasing NO content and antioxidant enzyme activity (SOD and GSH-Px), thus reducing ROS and malondialdehyde levels.

In another groundbreaking study, Li et al. explored the dual inhibitory effects of a commercial casein hydrolysate (CH) on dipeptidyl-peptidase IV (DPP-IV) and ACE, targeting anti-diabetic and antihypertensive properties. The study revealed intense activity against DPP-IV and ACE. In cellular experiments with human intestinal Caco-2 cells, CH significantly reduced DPP-IV and ACE activities by 61% and 77% after 6 h of treatment, underscoring CH's potential as a natural, side-effect-free alternative for managing hypertension and type II diabetes.

Innovative research by Gu et al. explored the preparation and properties of a Hericium erinaceus peptide-calcium chelate (HP-Ca), utilizing the mushroom's rich protein content and calcium-binding amino acids. Papain hydrolysis achieved optimal calcium-binding rates, resulting in chelates containing 6.8% calcium. Characterization confirmed HP-Ca's porous structure and calcium chelation through interactions with peptides' acidic amino acids and amide groups. HP-Ca showed remarkable stability against gastrointestinal digestion and significantly enhanced calcium absorption in Caco-2 cells, presenting it as an exceptional calcium supplement. This study illuminates the potential of Hericium erinaceus in developing bioavailable calcium supplements, offering a novel solution for calcium deficiency and bone health support. The increasing focus on exploring soybean, moth bean, skipjack tuna roes, and casein, along with recent studies on Gracilariopsis lemaneiformis (3), pearl matrix protein (4), and faba bean (5), milk (6) highlights the significant potential of bioactive peptides in improving human health.

In conclusion, bioactive peptides derived from various food sources are at the cusp of revolutionizing health and nutrition science. Their potential to serve as functional ingredients for managing hypertension, cardiovascular diseases, and other health conditions has been demonstrated, highlighting their value in developing therapeutic and dietary products. Generated primarily through enzymatic hydrolysis, fermentation, and gastrointestinal digestion, these peptides are vital to unlocking novel bioregulatory mechanisms and health benefits (Figure 1). However, the field is still nascent, with much to be explored regarding its structural characteristics, mechanisms of action, and optimal utilization methods. Future research directions should focus on large-scale studies to thoroughly understand these aspects, paving the way for discovering new bioactive peptides with diverse health roles. Such endeavors will broaden our knowledge and contribute significantly to public health improvement, emphasizing the importance of bioactive peptides in the next wave of nutritional and therapeutic innovations.

**Figure 1:**
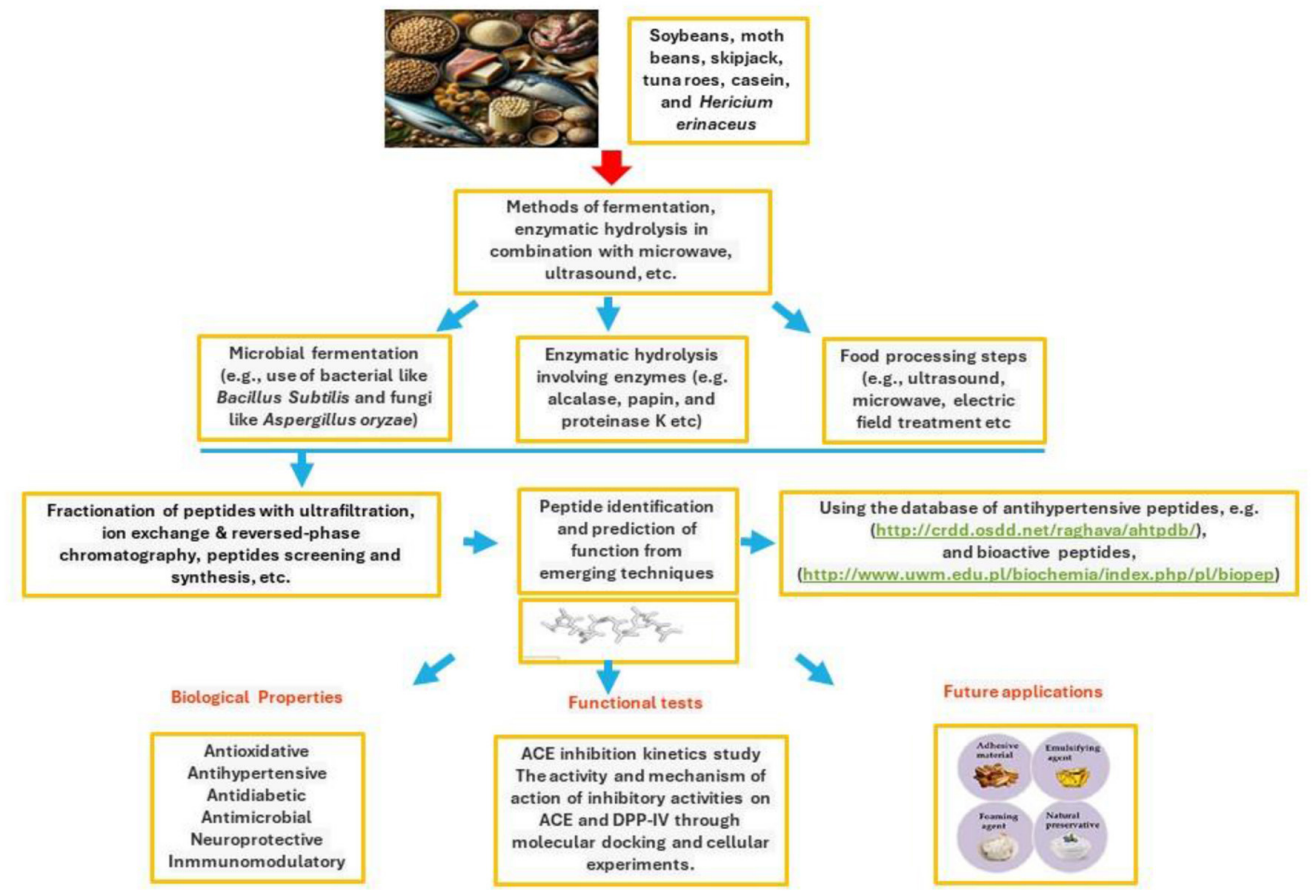
Isolation, identification and functional applications of peptides derived from beans, tuna, cheese, and mushroom.

## Author contributions

SZ: Writing – original draft, Writing – review & editing. XH: Writing – review & editing. LL: Writing – review & editing.
